# Part III. Analecta

**Published:** 1827-10

**Authors:** 


### PART III.

### ANALECTA.

### THE ROYAL COLLEGE OF PHYSICIANS,

As it was, as it is, and as it ought to be.

#### 1. CHARTER OF THE COLLEGE OF PHYSICIANS.

"Periturae parcite Chartæ."—*Juvenal.*

The following original and translation of this celebrated Charter (under the authority of which the College is now proceeding against a Graduate of Edinburgh—Dr. Harrison) will, we hope, be acceptable at the present moment.

#### CHARTER OF INCORPORATION.

ORIGINAL.

TRANSLATION.

Henry by the grace of God, King of England and France and Lord of Ireland, to all to whom these presents shall come, Greeting.—Inasmuch as we consider it to be the duty of our kingly office to consult in every way the happiness of those who are subject to our sway, and as this object would be most effectually attained by *putting a seasonable check to the practices of the wicked, we have judged it particularly necessary to repress the audacity of bad men, who profess medicine more from avarice than from conscientious and laudable motives, whereby divers injuries are done to the ignorant and credulous people*—We, therefore, partly following the example of well-regulated cities in Italy and many other countries, and partly yielding to the entreaties of grave and learned men, to wit, John Chambre, Thomas Linacre, and Ferdinand de Victoria, our physicians, and of Nicholas Halsewell, John Francis, and Robert Yaxley physicians, but principally of our most Rev. Father in Christ, Thomas, intituled the Lord Cardinal, presbyter of the holy Roman church of St. Cecilia beyond the Tiber, archbishop of York and most dearly beloved Chancellor of our kingdom of England, do will and ordain, that there shall be established a perpetual college of grave and learned men, who may publicly practise medicine in our city of London and its suburbs, and within seven miles of that city in every direction; and we trust that these, for their own credit, and for the public good, *will take care, as well by their own weight and example, to discountenance the ignorance and the rashness of the before-mentioned evil-disposed persons, as to punish them by our laws lately promulgated, and by the regulations to be made by the same College*; and in order that this may the more easily be accomplished, we have granted to the aforesaid Doctors John Chambre, Thomas Linacre, Ferdinand de Victoria, our physicians, and Nicholas Halsewell, John Francis, and Robert Yaxley, physicians, *that they and all men of the same faculty, of and in the aforesaid city, shall become in fact and in name one body, and perpetual commonalty or college, and that the same commonalty or college may every year, for ever, elect and make out of that commonalty* some man prudent and skilful in the faculty of medicine, for president of the same college or commonalty, to superintend, take cognizance of, and govern for that year the aforesaid college or commonalty, and all men of the same faculty and their business; and that the same president and college or commonalty shall have perpetual succession, and a common seal to be used in the affairs of the said commonalty and president for ever; and that they and their successors for ever be persons capable of purchasing and holding, in fee and perpetuity, lands and tenements, rents, and other property whatsoever.

We have also granted to them and their successors, for ourselves and our heirs, that they and their successors may purchase to them and their successors, as well within the aforesaid city as without, any lands and tenements whatever, not exceeding the annual value of *twelve pounds*, the statute of alienation in mortmain notwithstanding; also that they, by the names of the *President and College or Commonalty of the Faculty of Physic* in London, may sue or be sued, before any judges, and in any courts or causes whatsoever; and that the said President and College or Commonalty, and their successors, may hold lawful and honest meetings among themselves, and make rules and regulations for the wholesome government, superintendence and correction of the aforesaid College and Commonalty, and of all who exercise the same faculty of medicine in the said city, or within a circle of seven miles round the same, when, and as often as occasion shall require, lawfully, with impunity, and without hindrance from us, our heirs or successors, justices, escheaters, sheriffs, or other the bailiffs or servants whatsoever of us, our heirs, and successors. Also, we have granted to the same President, and College or Commonalty, and their successors, *that no one shall practise the said faculty within the said city, or within seven miles round the same, unless admitted by the said President and Commonalty, or their successors for the time being, by letters sealed with the common seal of the same President and College, under the penalty of one hundred shillings for every month in which he shall have practised the same faculty without such admission, one half thereof to be applied to the use of us and our heirs, and the other half to the use of the said President and College.*

Moreover, we will and grant, for ourselves and our successors (as far as in us lies) that there be elected every year, by the President and College of the aforesaid Commonalty for the time being, and their successors for ever, four persons, who shall have the superintendence and scrutiny, correction and government of all and singular the physicians practising the faculty of physic in the same city, and of other physicians whatsoever residing without the same, using or exercising the said faculty of medicine in any manner within the same city and the suburbs thereof, or within seven miles round the same, and also the punishment of them for their faults in the improper exercise and practice of the said faculty, and also the superintendence and examination of all sorts of medicines and the prescriptions of them by the aforesaid Doctors, or any of them, to be given, administered or applied to our liege subjects, for the healing and curing of their infirmities, when and as often as occasion shall require, for the advantage and use rum; ita quod punitio hujusmodi medicorum utentium dicta facultate medicinæ, sic in præmissis delinquent' per fines, amerciamenta, & imprisonamenta corpor' suor' & per alias vias rationab' & congruas exequatur.

of the said lieges, so that the punishment to be inflicted on any of the said practitioners in medicine, offending in the premises, shall be by fine, imprisonment of their bodies, and other reasonable and proper means.

We also will and grant, for ourselves, our heirs and successors (as far as in us lies) that neither the President, nor any of the aforesaid College of Physicians, nor their successors, nor any of them, exercising the said faculty within our said city and the suburbs thereof, or elsewhere, shall be summoned, or put upon any assize, jury, inquest, inquisition, attaint or other cognizance hereafter to be taken within the said city and its suburbs before the mayor and sheriffs or coroners of our said city for the time being, or to be summoned by any officer or servant, officers or servants, although the same juries, inquisitions or cognizances may have been lawfully summoned by the writ or writs of us or our heirs, but that the aforesaid master or governors and commonalty of the said faculty, and their successors, and each of them exercising the same faculty, be as against us, our heirs and successors, and against the mayor and sheriffs of our said city for the time being and all their officers and servants thereof wholly acquitted and discharged for ever by these presents.

TUNSTALL."

Provided always, that these our letters, or any thing in them contained, shall not operate to the prejudice of our said city of London, or the liberties thereof. And this is to be without fine or fee to be made, paid or anywise rendered to us for the premises or the sealing of these presents, any statute or ordinance or act at any time heretofore made, published, ordained or provided to the contrary thereof in anywise notwithstanding. In witness whereof we have caused these our letters to be made patent. Witness myself at Westminster, the twenty-third day of September, in the tenth year of our reign.

BY THE KING HIMSELF,

And by the authority of Parliament above given.

TUNSTALL.

Not being versed in law matters, we shall abstain from much remark on the legality or illegality of the power exercised by the College over those who practise physic in London and vicinity—leaving this to be decided by the proper tribunal in the approaching trial.

We think it will not be denied by any party, that a charter, a statute, or a bye-law, which is to operate on the science of medicine, and, consequently, to influence the fate of the people, as far as their health and lives are concerned, should have some foundation in civil rights—some colouring of natural justice—some support from common sense. It is by these principles alone that we shall presume to examine the policy, the spirit, and the operation of the above instrument, and the bye-laws growing out of it.

1mo. The charter was granted *ostensibly*, and, we hope, *bona fide*, for the very laudable purpose of "putting a check to the practices of the wicked," and "to repress the audacity of bad men, who profess medicine more from avarice than from conscientious motives, &c." For this purpose, six eminent physicians were ordered to enrol themselves, "*and all men of the same faculty*" in and about London, so as to constitute "a perpetual College of grave and learned men," for the guidance and regulation of themselves and all others, practising within the said circle.* At the time this Charter was granted, we do not see how the above desirable objects could be better attained than by the very step that was taken. Nay, were the Charter now to start into existence, for the first time, we defy human ingenuity to point out a better plan than that which Henry the Eighth, or rather his "dearly beloved Chancellor," devised for the regulation of the profession of physic —with this important exception, however, that the said Charter violated all the three principles of equal rights, natural justice, and common sense, by limiting the salutary operations of the College to London and seven miles around. Can any thing be more absurd, more unjust, more contrary to plain common sense, than that a law should protect the inhabitants of Putney or Barnes' Common from all ignorant pretenders and quacks, while the lives and health of the inhabitants of Richmond should be left a prey to "the audacity of bad men," and the delusive promises of unprincipled Charlatans?

To our understandings, nothing can be more clear than that the spirit and intention of the original Charter contemplated—nay, enjoined the general association of *all* regularly and properly qualified physicians, to become, "in fact and in name, one body and perpetual commonalty," by which association, "ignorance was to be discountenanced," "evil-disposed persons to be punished," and laws and regulations made for the respectability of the profession and the good of society at large.

2ndo. We believe it is an undisputed principle, that all regulations made subsequently to those of the Charter itself—namely, the bye-laws, should be in accordance with the spirit and intentions of the original Charter. This being granted, how are we to view the present law which limits the fellowship to physicians educated at Oxford and Cambridge—and excludes from all *commonalty* and participation in the affairs of the College, those "grave and learned men" who may have carefully studied, and regularly graduated in the first universities for medical education which the United Kingdom or Europe itself can produce? The three principles by which we are governed in our remarks—equal rights, natural justice, and common sense, give it decidedly against this exclusion;—the legality or illegality of the measure

---

* "Quod ipsi, *omnesque homines ejusdem Facultatis* de et in civitate prædicta, sint in re et nomine *unum corpus et communitas perpetua, sive collegium, &c.*"—*See Charter.*

must be decided elsewhere. That a small association of men educated at two universities, where no medical instruction is even professed to be given, and who may become fellows of the College without shewing a single document to prove that they ever attended a medical lecture beyond the walls of their *alma mater,* should thus be placed as masters over those who have gone through all the discipline of the Continental or the Edinburgh universities, is a thing most incongruous to common sense and natural justice.

3tio. That the exclusive privileges, honours, powers, or whatever the advantages may be, of the Fellows of the College, must always tend to unite them closely into one body, and render them anxious to enhance the dignity, the reputation, and the interests of that body, is a principle of human nature which has universally operated from the beginning of time—and which is by no means dishonourable to human nature. But what must be the effect of this operative principle on the class of Licentiates, who are thus excluded from all participation in the honours or offices of the College, whether the exclusion be contrary to, or accordant with, the spirit and letter of the original Charter? Let common sense, a knowledge of mankind, and the history of the past be asked. These will tell us that the same principle which stimulates the Fellows, as a body, to promote their own reputation and respectability, must inevitably render them careless about the reputation and respectability of the Licentiates. Nay, looking at human nature as it is, rather than as it ought to be, we cannot but come to the conclusion that, however the Fellows of the College may, *individually,* wish well to the Licentiates, they must, as a corporate body, wish and strive to exhibit that corporate body to the public at the expense of the extra-social class. Is this conclusion supported by historical facts? Undoubtedly it is. The College have admitted and *permitted* Licentiates, whom they would no more admit or *permit* as Fellows, than they would put their right hands into the fire. The College will not press us for proofs of this assertion. They are as *recent* as they are *notorious.* The division of physicians into two classes, then, must unavoidably be productive of degradation to one of them, independently of the constant jealousy and ill-will, however concealed, which must operate on a class of men, equal in all other respects, but *degraded* (the word is strictly applicable) in the profession which they exercise, by the invidious distinction which is made between Fellows and Licentiates.

If it be said that the Charter has empowered the College to make such bye-laws as may be deemed promotive of the interests and respectability of medicine, and the good of society at large; and consequently that it was authorised to make a distinction between Fellows and Licentiates; we say that the said distinction is contrary to the spirit, if not the letter of the Charter—and what is more, that it is contrary to the three principles by which we hold ourselves to be governed in this commentary—civil rights, natural justice, common sense.

It certainly does appear to us to be contrary to the spirit and letter of the Charter—for surely the threat and penalty denounced against those illegal practitioners who acted in defiance of the College, can never be fairly construed as authorising two classes of physicians in a College which is distinctly stated to be "*in fact, and in name, one body.*" It is difficult to say exactly what was the character of those against whom the penalty was denounced, unless they obtained a permit from the College. Either they were, or they were *not* properly qualified physicians. If they were properly qualified, why *force* them to become members of the said College? Or, was it likely indeed that they would require any force? If, on the other hand, they were *not* properly qualified, would the payment of a sum to the College, and the grant of a licence render them proper physicians to be let loose on society? But be the import of the passage what it might, it is high time that the process of *licensing* physicians to practise, where they are ineligible to become Fellows of the College or commonalty of physic, should be abolished. The act of taking or giving a *licence*, where the physician is regularly educated, (and it ought not to be given under any other circumstance) is an act of self-abasement on the part of the *Licentiates*, and an exercise of power which is any thing but magnanimous on the part of the body which imposes the degrading title on a fellow-labourer in the work of humanity, and the field of science! Raise the tests and proofs of classical erudition and professional acquirements to the highest pitch which human talent can attain, if you please; but, these tests and proofs obtained, leave men *equal* and *free*, to rise or fall on the scale of eminence, by their future exertions, and the force of their abilities.

But it will be urged that a superior general education is secured to the physician by a residence at Oxford or Cambridge. Although this is by no means certain, yet, granting it to be the case, on what principle of justice to the public, or of common sense, does the College prescribe this superior education for the *minor* portion of physicians in London, and allow the *major* part to get their education where they please? If the College insisted on this preliminary education for *all physicians* within their jurisdiction, there would be some consistency in the measure; but when they limit this superior education to the Fellows of the College, it can only be to distinguish these from the Licentiates—in short, it can only tend to the aggrandisement of their own body, (a very small part of the profession,) and not contribute to the public good, or general respectability of physicians at large.

We should suppose that it is the actual possession of knowledge, or rather of learning, which constitutes the advantage of this superior education;—or is there any occult moral virtue in the air of Oxford or Cambridge, which renders the Greek, Latin, and mathematics of those places more conducive to the future acquisition and practice of the medical sciences?

The College will admit that the *medical* part of a physician's education is of at least as much importance as the *classical*. Yet the College does not prescribe any particular climate or latitude where *medicine* is to be studied. The candidate for collegiate honours may draw *medical* lore from London, Paris, Edinburgh, or any spot on the face of the globe, or he may take it all from books—but Greek, Latin, and the auxiliary sciences, must be acquired in two particular counties of England, or else they are of no use to the owner! The latitude which is thus given for the acquisition of the more important portion of a physician's education, clearly shews that the restriction imposed on the less important portion, is a mere instrument for limiting the number and raising the character of a small proportion of physicians in London and its vicinity, without any view to the general advantage of physicians or the public. That a good preliminary education is of the greatest importance to *all* physicians, we readily grant; but that the acquisition of this part of a physician's education should be more localised than his medical education, cannot be maintained on any principle of natural justice or common sense. That classical learning is confined to Oxford or Cambridge, is too absurd a proposition to be even broached. Can the College turn out a more *erudite* physician than Dr. Cooke, who never breathed the air of those ancient seats of learning? In fine, if the College consider a good classical education essential to the physician, they do all in their power to degrade the character of the Licentiate, by admitting him *without that qualification*—and if he possess it, no matter where obtained, they do him an injustice, not fairly sanctioned by their Charter, in shutting against him the door of the Fellowship. We defy the most subtle casuist to controvert these positions on any of the principles by which we argue—equity, justice, common sense. He can only shelter himself behind the shield of the *law*—if that really affords a shield on this point.

But if the spirit and original boundary of the Charter have been overstepped (and we believe they have) by the creation of this invidious distinction between the Fellows and Licentiates of a college or commonalty; let us inquire whether or not the spirit of this Charter has been maintained, and its injunctions complied with, on other points. The preamble states, that it is the duty of the kingly office "to consult in every way the happiness" of those who are subject to the mild sway of the Eighth Henry; and then avers that "this object would be most effectually attained by putting a seasonable check to the practices of the wicked"—and by repressing "the audacity of bad men, *who profess medicine more from avarice than from conscientious and laudable motives*." Unless language was given to man in order that he might conceal his thoughts, it is as clear as the sun at noon day, that the foregoing preamble *describes* and points out for collegiate chastisement, the Charlatans of the present hour. Has the College directed its powers against, and levied its penalties on, this wide-spread class of evil doers? No! Against whom then are these powers of the Charter pointed? Against the well educated and regularly graduated physician, on whose moral character, or professional qualification, no stain is even insinuated! How applicable is the celebrated satire of Juvenal on this occasion!

"Dat veniam Corvis, vexat censura Colombas."

The statutes of the College, in fact, are made instruments to —

"Clip the dove's wings, and speed the vulture's course."

Or is it possible that in all these chartered aspirations for the happiness of the subject—for the repression of audacity, avarice, and ignorance in unprincipled quacks, EGO et REX MEUS have "paltered with us in a double sense," and that the meaning of all this philanthropic effusion is simply this:—"On every regularly educated physician in London and its vicinity, you shall levy a tax of 57 pounds, for his licence—and if he refuse to pay, you shall prosecute him. *After* he has paid for his licence, he may do anything he pleases to degrade the character of his *Fellow-licentiates;* for in the support of that you can have little concern." We believe that no such double meaning was concealed in the preamble of the Charter; but that the penalites were really designed for those characters pointed out. Yet the lapse of time has certainly brought these penalties and prosecutions into operation against those whose characters are free from blemish, and whose medical qualifications are unquestioned; while the unprincipled Charlatan is permitted freely to assume the title of DOCTOR and practise on the lives and purses of His Majesty's subjects.

We think there will hardly be a difference of opinion, among all unprejudiced persons, respecting the existence of the evils which we have been endeavouring to point out; but there will be many opinions as to the remedies which ought to be applied for the cure. Our own opinions on this subject will probably carry little weight with either party; but nevertheless we are entitled to an opinion, and we will give it.

So far from wishing to see the Charter of the College abrogated, and its powers annihilated; we wish the *former* to be restored to its pristine simplicity and spirit—and the *latter* (the powers of the College) rendered completely operative. Surely the President and Fellows of the present day can run no risk in approximating the constitution of the College as closely as possible to the original Charter. Can they not safely, as did Linacre and the other physicians above-mentioned, call together all regularly educated and properly qualified physicians, so as to form a College or Commonalty that might have superintendence over all physicians in the United Kingdom? Can there be a doubt that, if they thus acted, the legislature would furnish them with ample powers for the suppression of quackery and the protection of learning and talent? As we said before, let the *minimum* of scholastic education and professional attainments be fixed at the very highest point the College may think fit—but the proofs produced, and the personal examinations gone through, let the candidate be *admitted,* bona fide, into the College, and not *merely permitted* to have his name enrolled on a list which degrades him in rank, and precludes him from any voice in the Commonalty to which he is said to be *admitted.** This procedure would, at once, give prodigious strength and moral influence to the College, while it squared precisely with the spirit and even the letter of the original Charter. Let the vile *licensing* system be for ever abolished. Every physician in the United Kingdom would eagerly press for admission into a College where the only criterion—the only essential requisite, was high endowment, classical, philosophical, and medical. Whereas, in the present state of the case, nothing but dire necessity can possibly induce any man to place his neck beneath the yoke—or suffer himself to be branded with the word *licence,* as if his very existence was *illicit* till he had bought his *permit* from a College that turns him out of doors the moment he has paid his money!

But as it is the nature of all corporate bodies to adhere tenaciously to what appears to be their own peculiar advantages, till forced into accordance with the wants and wishes of society at large, so we do not expect that the College will pay any attention to the exposition which we have made of the existing defects. Nevertheless, the sun will not more certainly rise in the East, than will the time come, and that very soon, when the change which we have contemplated must take place. The present President and many of the Fellows are, we have reason to think, well disposed to do every thing that is liberal; and happy will it be for the College, if the liberal party are not outvoted and thwarted on this important occasion. In that case, there is but one constitutional measure to pursue: the whole of the Physicians of Great Britain, Licentiates and all, should lay a full, fair, and energetic representation of the case before the legislature—praying, not for an abolition of the College Charter, but for a restitution of it to its original spirit and simplicity, together with an extension of its powers to the whole kingdom. It would be difficult to conceive on what grounds the College itself could object to this investigation of its present laws, as compared with the spirit of its original constitution—especially when the

* The proofs of medical acquirements should not be trusted solely to an examination, *viva voce.* There should be a double check, the production of authentic documents as to the place of study, and the time spent in medical discipline. Yet the proof of medical acquirements in the Fellow of the College is limited entirely to the examination, for the production of the Oxford or Cambridge degree is not even pretended to afford any proof of medical knowledge. If then the College trust to an examination, and to an *examination alone,* for proof of competent *professional* knowledge in the Fellow, why should it not trust to an examination as to scholastic learning in graduates of other universities? This is a most important point to be urged against the present system—and we believe it has never been urged before.

professed and the real object of the petitioner's prayer, was an extension of the Collegiate powers beyond their present circumscribed boundary:—and we venture to predict that, whenever this representation is made, and the matter properly scrutinized, the conclusions of the investigators will not be materially different from those which we have ventured to draw in the present paper, under the guidance of civil rights, natural justice, and common sense.

---

As the approaching trial between the College and Dr. Harrison, involves questions of the highest importance to the profession and to society at large —and as the issue of the contest is calculated to excite a more intense degree of interest throughout the profession than any thing which has occurred for many years past, we shall lay before our readers some extracts and documents which may enable them to meditate with more advantage on the judicial struggle which is about to take place between obsolete statutes and natural justice.

It appears by 14 and 15 of Henry VIII. c. 5, that the Charter of incorporation, (of which we have given the original and a translation) was confirmed by Parliament. The six physicians already alluded to, and "*all* other men of the *same faculty* within the City of London, &c." were incorporated "in *one body* and perpetual *commonalty or fellowship* of the faculty of physick." But in addition to this, the following clause was appended to the Act.

"And where that in Dioceses in *England*, out of *London*, it is not light to find alway Men able sufficiently to examine (after the Statute) such as shall be admitted to exercise Physick in them, that it may be enacted in this present Parliament, That no Person from henceforth be suffered to exercise or practice in Physick through *England*, until such time as he be examined at *London*, by the said President, and three of the said Elects; and to have from the said President or Elects, Letters Testimonial of their approving and Examination, except he be a Graduate of *Oxford* or *Cambridge*, which hath accomplished all things for his Form, without any Grace." 13.

We think that all impartial men will construe this statute as follows:— To become one of the Commonalty or College, so often mentioned, it is necessary for the Graduates of other Universities than those of Oxford and Cambridge, practising as physicians in the country, to appear before and be examined by the president and three of the elects—otherwise his practice is illegal. But the Graduates of Oxford and Cambridge may practise *in the country*, without any such examination. The advantage accorded to the Oxford and Cambridge University by this statute, is merely the exemption from examination for country practice. There is nothing in the Statute which can at all authorise the present distinction between Fellow and Licentiate—we mean the non-eligibility of regular Graduates of other Universities to become Fellows of the Commonalty or College of London.

After this we hear nothing more of any consequence till the reign of King James, when a series of College questions were resolved by the then Lord Chancellor and Judges. We shall extract a portion of these questions with the answers appended to them.

"Quest. 1. Whether Graduates of Oxford and Cambridge may practise in *London* or seven miles compass of the same without licence under the said College Seal, by virtue of the clause in the end of the Statute of 14 H. 8. and whether that clause hath not relation to the Statute of 3. H. 8. only, or how far it doth extend?

"Resp. All resolved, that no Graduate that is not admitted and licenced by the President and College of Physicians under their Common Seal, could practise in London or within 7 miles compass of the same.

"Quest. 2. Whether, by Graduates, Graduates in Physick onely are to be understood?——

"Resp. They resolved That the exception in the Statute of 14. H. 8. cap. 5. of Graduates in the two Universities, is to be understood onely of Graduates of Physick and of no others. And all resolved, That by that exception those Graduates may practise in all other places of *England* out of *London* and 7 miles of the same without examination; But not in *London* nor within the said Circuit of 7 miles.

"Quest. 3. If Graduates not admitted to practise in *London* practise there, whether, for evil practice or misdemeanor therein, they be not subject to the Corporation and Government of the College?

"Resp. They all agreed, That they are subject to the Government and correction of the College by an express Clause of the said Charter enacted which giveth to the President and College *Supervisionem Scrutinium, Correctionem & Gubernationem* as well of all persons using the practise of Medicine within the City &c.

"Quest. 4. If they may not practise without admission of the College (as their Letters Patent plainly import) Then whether such Graduates are not subject to the examination, without which there were never any admitted; and without which the admission cannot be approved; because every Graduate is not absolutely good *ipso facto*?

"Resp. It was resolved by all That all that practised or should practise Physick either in *London* or within the compass of seven miles of the same, must submit themselves to the examination of the President and College if they be required thereunto by their authority notwithstanding any licence, allowance or privilege given them in *Oxford* or *Cambridge* either by their degree or otherwise."* 95.

It is not till the reign of Charles, viz. in the year 1674, that we hear any thing of a distinction between Fellows and Licentiates, or of the exclusive right of Oxford and Cambridge Graduates to the Fellowship. This too is only in a Royal letter from the King to the College, without any appearance of parliamentary confirmation.

CHARLES R

Trusty and welbeloved wee greet you well

Whereas we have been informed That there are *several pretended Physicians & Doctors graduated in the Universitys beyond the Seas who by indirect means endeavour to be received into that our Royal Colledge as Honorary Fellows, without incorporation into either of our Universities, or previous Examination & approbation,* according as it is expressly required by y^e Statutes to y^e great prejudice of y^e ffellows of or said Colledge & their Successors & of the Priveledges & immunityes granted to them by or Royal predicessors & orself, Wee having taken the same into or Royal Consideration have thought fit *to signifye or pleasure* to you, & doe accordingly *direct you* not to admit any person whatever as a Fellowe of the Society & to enjoy y^e priviledges of or s^d Colledge that hath not had his Education in either of or Universityes of Oxford or Cambridge kept his Act for D^r in Physick & don his Exercises accordingly, or that is not encorporated & licenced there haveing first taken the Oathes of Allegiance & Supremacy, & haveing been by you afterward examined & approved of according to the Statutes. And to the Intent this

* Med. Jurisprudence, Vol. 3, p. 94.

or pleasure may be better observed wee doe likewise *hereby require you* to cause these or Letters to be entered upon the Registe of or said Colledge & so wee bid you ffarewell, Given at or Court at Whitehall Febr. 12th 1674 in the 26th year of or Reighn."

This document clearly proves, we think, that the Fellowship was not exclusively kept for Graduates of Oxford or Cambridge, previously, else why the necessity for this Royal Letter? It offers, we imagine, incontestible proof that no such distinction as that which now exists between Fellows and Licentiates, was contemplated by, or contained in the original Charter—hence the advantage of returning to the spirit of that ancient instrument.

This Letter sets out by artfully sounding an alarm that "several *pretended* physicians and doctors graduated beyond the seas," had endeavoured by *indirect* means to be received into the Royal College "as *honorary Fellows*," "without incorporation *or* (mind that) previous examination and approbation, &c." Now this *or*, instead of *and*, clearly shews that previously to this Royal Letter, Physicians had been received into the Commonalty or Fellowship, who had graduated beyond the seas, but who had submitted to examination and paid the fees. The Royal Letter therefore bears intrinsic marks of *secret influence* working on the Royal mind, in favour of a *restriction* which did not previously exist, and of which we see no indication in the original Charter—no confirmation by parliament following this loving letter. But whether it was confirmed by Parliament or not, the College cannot surely plead an edict or a law which they have violated and do violate whenever it suits their purpose. If it be good to break a law annually, one would suppose it still better to repeal it. Or is there some secret key to this Royal mandate, which indicates when it is to be obeyed and when disregarded? Are there certain *letters* in the alphabet or *combinations* of letters, to which the ROYAL LETTER sympathetically responds, or on which it repulsively frowns? We should suppose that this is the case. Thus the Royal Letter spontaneously opens its seal at the word "Sir James"—but is deaf to the word "Sir Gilbert." The letter B. followed by an A. dissolves King Charles' prohibition against those Doctors who have "graduated beyond the seas;" but if the letter B. is followed by an L. it is quite another affair, and the Royal VETO is in full force! The case is plain enough to common sense. The Royal injunction is a mere instrument or key by which the door of promotion or favouritism is opened or locked, according as inclination prompts or interest operates.

And granting that certain ultramarine doctors were endeavouring, (in the time of King Charles) *by indirect means*, to get themselves enrolled as honorary members of the College without university matriculation or personal examination and disbursement of the money, surely these things can be guarded against by the watchful eyes of our censors, without any necessity for deviating from the original spirit of the charter. Much do we suspect that the fears engendered in the royal breast respecting the honour of the College, arose from "information" tendered by people who kept a bright eye on their own interests rather than on the interests of the King's liege subjects.

We shall now lay before our readers some particulars of a remarkable trial that took place between a physician of the name of Bonham, and the College, which bears very close indeed on that which is soon to be exhibited in one of our own courts of the present day. It is extremely difficult to pick out much common sense or rational argument from the mass of technical absurdities and legal quibbles with which this trial abounds. We have therefore left a great portion of its Cymmerean verbiage to rot in the original rubbish where it lies. We shall, however, preserve some curious scraps from this legal dunghill, which may afford ample materials for serious reflexion, and some shrewd judicial observations which are little calculated to aid the cause for which this document has been so carefully preserved by the advocates of ancient, obsolete, and irrational statutes.

The said Thomas Bonham, a Doctor in Philosophy and Physick, of Cambridge, brought an action against the College Censors of his day, for seven days' imprisonment, in the 4th year of the reign of King James. The defendants pleaded the celebrated Charter of Henry VIII. especially two clauses beginning, "*quod cum Regii officii, &c.* (see the beginning of the Charter) and "preterea voluit, &c." (see the same) pointing out the parliamentary confirmation of the said Charter, in the 14th year of Henry the Eighth's reign. They further pleaded that the said Thomas Bonham, "exercebat artem medicinæ, *non admissus per literas præd' presidentis & Collegii sigillo, &c.** ubi revera prœd' Thomas Bonham fuit minus sufficiens ad artem medicinæ exercend?" Dr. Bonham then, a Graduate of the English Universities, being weighed in the scale, and found "*minus,*" was interdicted from practising by the President and Censors, and subsequently fined in the sum of five pounds for continuing to practise, in contempt of the College after rejection.†

Again Dr. Bonham was summoned and amerced in the sum of *ten pounds.* He was a third time summoned before the dread tribunal of censors, and asked if he would humble himself before the College for his disobedience, and submit himself to examination. But the Doctor appears to have been made of real opposition stuff; for he "answered, that he had practised and would practise physic, within London, *nulla a Collegio petita venia*—and that he would not submit himself to the President and Censors; affirming that the President and Censors had no authority over those who were Doctors in the University." Upon this, the Censors did forthwith incarcerate the body of the said Dr. Bonham, in the Compter of London!‡

It was natural enough for Dr. Bonham now to plead that clause of the

---

* This clearly shews that the graduates of Oxford and Cambridge were *admitted* by letters of the President, &c. in the same terms by which the Licentiates are *now* admitted—and consequently that the admission was the same for both at that time. This is a point of great importance, as proving that the innovation of dividing the College into two classes—Fellows and Licentiates, had no foundation in the original Charter.

† This fining and imprisonment, after failing in an examination, shews the difference between former and present times. What would have been the consequence if Dr. Armstrong and Dr. John Mason Good had been *fined and imprisoned* immediately after their rejection by the present College!

"Tempora mutantur, et nos mutamur in illis."

The College ought to bear in mind how dangerous a procedure it is to kindle up the exercise of Statutes at which science and sense *should* blush—and for which the College itself *may* perchance grieve!

‡ Dr. Harrison may thank his stars that he had a Henricus *Halford,* instead of a Henricus *Octavus,* to deal with; otherwise he might have passed the dog-days of 1827, in the Compter, instead of rolling in his carriage about Cavendish-square! Sincerely do we regret that such a man as Sir Henry Halford should be doomed to support such execrable laws! There is not a man in the United Kingdom whose liberal mind will more revolt against the execution of a statute which will be recorded in the latest annals of medicine, as a foul blot on the science and sense of the Nineteenth Century! But we have *more than hopes*—we have *strong expectations,* that,

Parliamentary Act (confirmatory of, and additional to the Charter) which expressly exempted the graduates of Oxford and Cambridge from the examination of the London College;—the said graduation at the English Universities having "*accomplished all things for his form, without any grace.*" He proved that he was a graduate of Cambridge, and consequently had "accomplished all things," until "the defendants (the Censors) had imprisoned him." But, alas! the meaning of words *on one side* of a question is quite different from the meaning on the *other side.* The case was often argued by the Serjeants at bar, and at last was argued by the Justices.

It appears that Justice Daniel was of opinion "that a Doctor of Physic, of the one University or the other, &c. was not within the body of the Act—and if he was within the body of the Act, that he was exempted by the said latter clause." By some legal logic, however, this was carried against Justice Daniel. Some observations of the celebrated Coke, then Chief Justice, are well worthy of record in this place, as shewing the absurdity of clinging to old statutes, that violate common sense and public justice. After paying a somewhat dubious compliment to the College Doctors, he remarked that—"no comparison was to be made between that private College and either of the Universities of Cambridge and Oxford, no more than between the father and his children, or between the fountain and the small rivers which descend from it. The University is *alma mater*, from whose breasts those of that private College have sucked *all their science and knowledge*, (which I acknowledge to be great and profound). The University is the fountain, and the like private Colleges are *tanquam rivuli.*"

If a man were to preach the doctrine now that the Fellows of the College drew all their knowledge from Oxford or Cambridge, he would stand a fair chance of being prosecuted for a libel by the said Censors; yet this was the grand argument made use of formerly for keeping up the privileges of Oxford and Cambridge. All claim on the part of medical knowledge is now dropt by the supporters of the Universities; but then the Latin, Greek, Mathematics, and *Moral* Philosophy of the Universities are of such a kind as to make the future physician pre-eminent in the knowledge of diseases, their causes, and their remedies, beyond the scholastic lore of all other places whatever.

But to return. The Judge properly observes that the laws of the College, as granted by the Charter, were evidently directed against five classes of people, namely the *Improbi*, the *Avari*, the *Malitiosi*, the *Temerarii*, and the *Inscii*. These were to be prosecuted, fined, or confined by the College. We ask the College if it was ever contemplated, or if contemplated by the Charter, was it right, that any of the above characters should be *admitted* as *Licentiates* by paying a fine to the College? Certainly not. It is therefore most disingenuous to consider the class of Licentiates as taking their origin from this farrago of Charlatans. On the other hand, let us see who those are whom the Chief Justice points out as fit to be admitted into the Commonalty of Physicians. They are—" 1. *Profound.* 2. *Sad.* 3. *Discreet.* 4. *Groundly learned.* 5. *Profoundly studied. And it was well or-*

---

under this liberal and enlightened President, the original Charter of the College will, ere long, start into a new existence, divested of the narrow prejudices and illiberal shackles, with which poor, weak, jealous, and short-sighted man has debased and dishonoured a noble and Royal Charter. Never was a fairer field for acquiring immortality opened, than Sir Henry has now before him; and we are much deceived in his character, if he do not take advantage of the golden opportunity. It may never again occur!

*dained that the Professors of Physic should be profound, sad, discreet, &c. and not youths who have no gravity and experience.*"* Is there any allusion here to the Graduates of Oxford and Cambridge as the only Physicians proper for the Association? Not the slightest idea of the kind appears to enter the mind of Chief Justice Coke. It is true that he considers it a matter to be presumed that the Graduates of Oxford and Cambridge should have the above qualifications, by virtue of their education; but he no where insinuates that these *only* are to be Fellows of the College. His words are :—"And it ought to be presumed, every Graduate of *any of the Universities* to be within the statutes, viz: to be profound, sad discreet, &c."

The judges determined that the Censors had no power to *confine* Dr. Bonham, because he belonged to the last class described; but, after a long discussion, they determined that Dr. Bonham was finable by the College, although a graduate of that *Alma Mater* from whence the said College Alumni "*had sucked all their science and knowledge*," although the statute had expressly declared that the said graduation at Oxford or Cambridge "accomplished *all things* without grace"!! Such are the absurd interpretations of statutes enacted without regard to common sense, public utility, or rational justice! But this is not all. These men of wisdom on the bench decided that, in the first place, "he who practises physic in London in a good manner, although he doth it without a licence, yet it is *not any prejudice to the body of man*." He may practise thus for the space of 27 days; but if he write a prescription on the 28th day, were it to save the life of a King or a Prime Minister, he is actionable, and must be prosecuted as the Act directs !†

"And the law hath *great reason* in making this distinction; for divers nobles, gentlemen, and others, come upon divers occasions to London, and when they are here they become subject to diseases, and, thereupon, *they send for their physicians in the country*, who know their bodies, and the cause of their diseases, (causes arising in London!) Now it was never the meaning of the Act to bar any one of his own physicians; and when he is there, *he may practice and minister to another by two or three weeks, without any forfeiture*."—*Paris, vol.* iii. *p.* 108.

To shew that the College has power, and did formerly exercise it, over irregular and ignorant practitioners, we shall here cite the case of Groenvelt, who was fined and *confined* by the College, and who brought an action against the College, "*for trespass, battery, wounding, and false imprisonment*." The Censors, or defendants, pleaded, as usual, the Charter, by which they were authorised to amerce and imprison those who did not properly and skilfully exercise the healing art. They averred that the said Groenvelt did, on the 1st of January, in the eighth year of King William's reign, administer "*bad and unwholesome physick to one woman, and that the said woman and her husband complained to the defendants, being the Censors of the said College*." The Censors summoned the delinquent before them, and, "upon examination, they found him guilty of administering unwholesome physick, by means of which the said woman languished"—but, fortunately, did not die! For this grievous offence, they fined the poor Doctor £20. and then incarcerated his body! For this the action was brought against the College; but Chief Justice Holt supported the Censors of the College, and considered them as fully empowered to act as they did. The Judge laid it down as good law, that the Censors are constituted by the statute, "judges of the fact, what *is* a mal-administration of medicines,

* Paris, Med. Jurisprudence, Vol. iii. Appendix I.
† Vide Paris' Med. Jurisprud. vol. iii. p. 108.

and what is not: and they are judges of record, for they have authority to impose fine and imprisonment." It is hardly necessary to say, that poor Groenvelt was nonsuited.

Now, as the statutes confirmatory of the Charter have not been repealed, it is evident that the College possesses at least as much power over ignorant or quack doctors, as they possess over skilful and regular physicians: and if so, why do they not exercise this power? In fact, they possess much more power over the charlatan, than over the *alieni homines*, the graduates of Scotland, for example; because they can imprison, "pro delictis in *non bene* exercendo, &c." whereas they can only fine the regular physician, who does not, of course, come under the *non bene exercendo* clause. There can be no doubt, however, that they would punish, *by expulsion*, any FELLOW of the College, who did any thing to disgrace their own body; but a LICENTIATE, as an outcast from the College, is under little or no control, except from the dictates of his own conscience. He has no reward to expect from the College for merit—no reprehension for deviations from strictly professional and ethical conduct—if we may judge by some recent exhibitions and public correspondence.

As it is our intention to prosecute this enquiry through all its ramifications, we shall here conclude this first article, already too far extended. From the letters which have passed between the Censors of the College and Dr. Harrison, it is evident that both parties are now pledged to bring the matter in dispute to a legal issue. Whichever way the verdict may go, we are convinced that much good will result to the profession, and society at large. The execution of a statute which violates common sense and outrages public feeling, will be sure to excite an investigation, and lead to an enquiry that must ultimately abrogate the unnatural statute, or at least nullify its operation. We shall, therefore, wind up this article with a sentence from Chief Justice Coke's observations, which will probably not be lost in a certain quarter.

"And it appears in our books, that, in many cases, the *common law* will controul Acts of Parliament, and adjudge them to be utterly void: for, when an Act of Parliament is against common right or reason, and repugnant to be performed, the common law will controul it, and adjudge such Act to be void."*

If the above opinion, which we hope is prophetic, was ever applicable at any period, it is entirely so at the present moment. But, in our next, we shall return to the subject of reviving the original Charter, and extending its useful powers—concluding as we began,

"Perituræ parcite Chartæ.—Juv.

* Paris, Med. Jurisprud. trial of Dr. Bonham.

